# Feasibility and Validity of Postural Reaction Tests in the Neurological Examination in Healthy Rabbits

**DOI:** 10.3390/vetsci10030176

**Published:** 2023-02-22

**Authors:** Chyong-Ying Tsai, Pin-Huan Yu, Wei-Hsiang Huang, Ya-Pei Chang

**Affiliations:** 1 Graduate Institute of Veterinary Clinical Science, School of Veterinary Medicine, National Taiwan University, Taipei 106319, Taiwan; 2 Section of Small Animal Surgery, National Taiwan University Veterinary Hospital, National Taiwan University, Taipei 106328, Taiwan; 3 Graduate Institute of Molecular and Comparative Pathobiology, School of Veterinary Medicine, National Taiwan University, Taipei 106319, Taiwan

**Keywords:** rabbit, neurological examination, postural reaction, hopping reaction, feasibility, validity

## Abstract

**Simple Summary:**

Neurological examination is crucial for clinicians when animals present neurological symptoms. Rabbits, as prey animals, can be easily frightened and may not react as anticipated during examinations. Most of the literature describing how to perform neurological examinations in rabbits is limited to personal experience, with only one recent study evaluating neurological examinations in healthy rabbits. However, some tests were not included in that study, and our clinical experience differed from some of their conclusions. The current study focused on postural reaction tests and evaluated whether the rabbit was willing to cooperate with each test and the normal response rate for each test/method. In addition, the response rates of tests/methods with similar neuroanatomical pathways were compared. In total, 34 clinically healthy rabbits were enrolled in this study. Our results demonstrate that the response to postural reaction tests is highly variable in healthy rabbits. Moreover, the hopping reaction (by holding and lowering the rabbit quickly towards the floor with only the tested limb touching the ground), hemi-walking, wheelbarrowing, and righting response are tests likely to elicit consistent and normal reactions.

**Abstract:**

Neurological examination remains a fundamental step in the care of patients with neurological disorders. However, studies evaluating the feasibility and validity of neurological examination in rabbits are limited. In this study, postural reaction tests or methods commonly performed in dogs and cats were evaluated in clinically healthy rabbits, and we aimed to postulate a simplified examination list according to the results. The feasibility and validity of each test were determined and screened using a cut-off value of 90%. For the remaining tests/methods, the response rates of tests with similar neuroanatomical pathways were compared. Among the 34 healthy rabbits tested, one method of the hopping reaction (holding then lowering the rabbit quickly towards the floor with only the tested limb touching the ground), the hemi-walking test, the wheelbarrowing test, and the righting response yielded a feasibility and validity of over 90%. When comparing tests/methods with similar neuroanatomical pathways, the normal response rate of the hopping reaction was comparable to that of the hemi-walking test. We conclude that in healthy rabbits, hopping reaction tests using the method mentioned above, hemi-walking, wheelbarrowing, and righting responses are likely to be feasible postural reaction tests that yield consistent and normal responses.

## 1. Introduction

Neurological examination is essential for clinicians to determine the neuro-localization of lesions. However, rabbits are prey animals that may not react as anticipated during an examination or under stressful conditions. In our experience, even rabbits without neurological impairment may freeze and/or display neurological deficits during an examination. Therefore, clinicians should interpret the results of neurological examinations with great care.

Various textbooks and the literature have described how to perform neurological examinations in rabbits. However, most of them are limited to personal experience, and suggestions vary among authors [1,2,3,4,5]. The neurological exams were recently assessed by Warnefors et al. in healthy rabbits, regarding the possibility of practical performance of each test and whether an expected response was obtained [6]. Individual tests in the categories of cranial nerves, postural reactions, and spinal reflexes were assessed, and a shorter, modified version of the neurological examination was postulated. In the cranial nerve category, the menace response elicited a successful neurological response in none of the rabbits; therefore, it was excluded from the final version. Patellar, withdrawal, and perineal reflexes were included in the spinal reflex category. For postural reactions, tests in the final version included conscious proprioception, wheelbarrowing, hemi-walking, placing, and righting reactions. Our clinical experience with performing cranial nerve examinations and spinal reflexes in rabbits is similar to that of Warnerfors. However, in the postural reaction category, we noticed that some tests with a low success response rate remained in the final version, and the criteria for selecting individual tests were not clearly described. Moreover, all the tests were performed using only one specific method, and some tests (such as the hopping test) were excluded based on their pilot study.

In the literature on small animals, different methods of performing individual neurological tests are sometimes mentioned, especially when encountering feline subjects, who are more prone to react abnormally even without clinical neurological dysfunction. Quitt et al. proposed and compared three methods of performing the menace response and tested them in healthy cat subjects [7]. For the cutaneous trunci muscle reflex, the most common method is to pinch the skin of the dorsum using hemostatic forceps. Foss et al. and Tsai et al. proposed several alternative methods and compared their performance with that of the original method in healthy cats [8,9]. In dogs and cats, the hopping response is usually tested by lifting and holding one limb of the animal such that most of its weight is supported by the opposite limb, then pushing the animal laterally [10]. However, in our experience, some cats, particularly nervous cats, may display a delayed or absent hopping response when examined using this method. Garosi recommended that if a cat is hesitant to hop, it should be held with three limbs restrained, lowered abruptly to the ground surface with the tested limb extended and then moved laterally to force it to hop on that limb [11].

Postural reactions require the function of proprioceptive and motor systems, which generally involve the entire nervous system [10,12]. Lesions affecting any component of the sensory or motor system can lead to abnormal responses. Therefore, testing postural reactions is crucial for detecting subtle neurological deficits that may appear before any detectable abnormality in gait is observed [10]. As our clinical experience with postural reactions in rabbits varies from the conclusion of Warnefors et al., this study aimed to assess the feasibility and validity of postural reaction tests in clinically healthy rabbits from the Taiwanese rabbit population. In addition, several alternative methods for testing hopping reactions have been evaluated. A simplified examination list of postural reaction tests was suggested based on our results.

## 2. Materials and Methods

### 2.1. Animals

Two sources of animals were enrolled in this study: rabbits owned by staff or students of the National Taiwan University Veterinary Hospital, and rescued rabbits from the Taiwan Rabbit Saving Association. The study was conducted between September 2014 and May 2015. The Institutional Animal Care and Use Committee (NTU-104-EL-00076) approved the study protocol. Informed consent was obtained from the owner or the Taiwan Rabbit Saving Association of all animals described in this study for all procedures undertaken.

The inclusion criteria were age >6 months, good general health, and no previous neurological disease. Health status was determined based on observations from the owner or caregiver and a complete physical examination performed by the author before the neurological examination. Animals were excluded if they were lethargic, anorexic, underweight, dehydrated, or had ophthalmologic, orthopedic, or marked dermatological paw disease based on the recent history and findings from the physical examination. The breed, age, sex, neuter status, and location of performing the neurological examinations were recorded.

### 2.2. Postural Reaction Tests in the Neurological Examination

**Figure 1:**
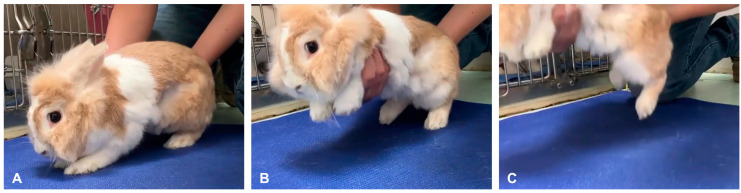
Three different methods to test the hopping reaction in the left pelvic limb. (**A**) Holding the animal with its 3 feet on the surface. (**B**) Holding the animal with only the tested limb touching the surface. (**C**) Holding then lowering the animal quickly towards the floor with only the tested limb touching the ground.

A single examiner (CYT) performed a complete neurological examination in the same order for all rabbits. The examination was initiated by the inspection of mentation and posture, followed by cranial nerve assessment, postural reaction testing, and spinal reflexes. Postural reaction tests were conducted in the following order: proprioceptive positioning, three methods of hopping reaction, hemi-walking, wheelbarrowing, extensor postural thrust, tactile placing, and righting response. The methods used for individual postural reaction tests are described in Table 1. Three methods for testing hopping reactions are illustrated in Figure 1. The feasibility of each test was recorded. The result was recorded as “unfeasible” if the rabbit was unwilling to cooperate with a particular posture or could not be restrained during the test. If the test was deemed “feasible”, the response was recorded as normal or abnormal. Each test was repeated consecutively three times and recorded for further analysis. This was designed to resemble the scenario in which clinicians may repeat a specific test several times when assessing neurological function to avoid misleadingly decreased or increased responses that may occur due to influences from the animal’s posture or unintentional movements, or improper positioning of the subject [12].

### 2.3. Data Analysis

As this study aimed to suggest a simplified list of postural reaction tests in rabbits based on feasibility and validity, individual tests/methods were screened and compared at several stages.

The first stage screened the tests/methods based on feasibility. The tests/methods were eliminated from further analysis if the feasibility of the population was less than 90%. In the second stage, the remaining tests/methods were screened based on their validity. As we aimed to evaluate the validity of individual tests/methods in healthy animals, more stringent criteria were used. We assumed that healthy rabbits without neurological diseases would display a normal neurological response. The results of the three attempts for each test were taken together for categorization. If the responses were normal three times, a score of zero was assigned. If the responses were other than normal (including absent, decreased, increased, and exaggerated responses) in either attempt, a score of 1 was assigned. The normal response rate for each test performed on the left or right side of the subject was compared using McNemar’s test. The side with the significantly lower response rate was recruited for further analysis. If there was no significant difference, the results from the left side were used for further comparison. Tests/methods with a normal response rate of ≥90% were selected as potential items in the simplified list of postural reaction tests in rabbits.

The third stage of the analysis compared tests/methods with similar neuroanatomical pathways in pairs, if they passed the previous two stages. The designed pairs included proprioceptive positioning versus tactile placing tests, methods of hopping reactions, and hopping versus hemi-walking reactions. Using McNemar’s test, a test/method with a higher normal response rate was chosen for the final simplified exam list.

All statistical analyses were performed using SPSS version 25.0 (IBM Corp., Armonk, NY, USA). Statistical significance was set at *p* < 0.05.

## 3. Results

Thirty-four rabbits were included in this study, representing ten breeds (eleven Dutch, six New Zealand White, three Netherlands Dwarf, three English spots, two lion head rabbits, one Crème dArgent, one Dwarf Hotot, one Polish, one Champagne Argente, and five mixed breeds). The mean age was 2.4 years, ranging from 0.5 to 11 years. There were 15 intact females (44.1%), 8 neutered females (23.5%), 5 intact males (14.7%), and 6 castrated males (17.6%). The examination was performed in an examination room at the National Taiwan University Veterinary Hospital in 8 rabbits (23.5%) and at the Taiwan Rabbit Saving Association in 26 rabbits (76.5%).

In the first stage, the extensor postural reaction test yielded a feasibility of only 76%; hence, it was eliminated (Table 2). The proprioceptive positioning test performed on the pelvic limbs was also excluded, with a feasibility of 85%. No difference was observed in the normal response rate between the left and right sides for any of the remaining tests. Data obtained from the left side were used for further analysis of all the above tests.

In the second stage, tests/methods yielding a normal response rate of ≥90% included method C of the hopping reaction, hemi-walking test, wheelbarrowing test, and righting response (Table 2). The tactile placing test in the pelvic limbs produced an “absent response” in all subjects. Other tests/methods yielded variable proportions of decreased and absent responses.

For the third stage, a comparison between proprioceptive positioning and tactile placing tests was not conducted because of the low validity of these tests (eliminated in the second stage). Among the three methods of the hopping test, only method C passed the second stage and hence was compared with hemi-walking reactions. The normal response rates for the two tests were 97% (32/33) and 91% (30/33), respectively. No significant difference was detected between the normal response rate of hopping reaction method C and the hemi-walking test (*p* = 0.6). Therefore, both tests were selected on the basis of the exam list. In summary, based on our results, the final simplified exam list included method C for the hopping reaction, hemi-walking test, wheelbarrowing test, and righting response.

## 4. Discussion

In this study, we demonstrated that the feasibility and normal response rate of postural reaction tests were highly variable in clinically healthy rabbits. The righting response was normal in all healthy rabbits willing to undergo manipulation for this test. The hopping reaction (performed by holding and then lowering the animal quickly towards the floor with only the tested limb touching the ground), hemi-walking test, and wheelbarrowing test were feasible and yielded normal response rates ≥90%.

The difficulty of performing neurological examinations in rabbits has been mentioned in many textbooks, and various personal experiences have been reported. Obtaining accurate lesion localization in this species may be difficult because unexpected responses may lead clinicians to incorrect conclusions. Our results support this impression, as many tests commonly applied in dogs and cats, such as proprioceptive positioning, performed poorly when tested in healthy rabbits.

In a previous study, the practical performance and expected response of individual neurological tests, similar to the feasibility and validity of our study, were assessed in 26 healthy rabbits [6]. These included various tests in the categories of general observation, cranial nerves, postural reactions, and spinal reflexes. However, the hopping test was excluded based on a pilot study conducted on three rabbits, as the authors deemed that its diagnostic value was not proportional to the stress induced in the subjects. As a result, they proposed a shortened neurological examination list, and included conscious proprioception, wheelbarrowing, hemi-walking, placing, and righting reactions as suggested postural reaction tests. In addition to the tests evaluated in Warnefors’ study, we included the hopping response and extensor postural thrust, which are commonly performed in neurological examinations in dogs and cats. The hopping response was readily performed in all but one subject, without resistance or excessive struggle. Furthermore, method C (hold, then lower the animal quickly towards the floor with only the tested limb touching the ground) produced a 97% normal response rate in both the thoracic and pelvic limbs. In contrast to Warnefors’ study [6], we conclude that performing the hopping response appropriately can yield considerably high normal response rates in neurologically healthy rabbits. However, it should be noted that proper rabbit handling techniques are essential when performing neurological examinations, particularly when lifting the rabbit [13].

Compared with the previous literature, our results are mostly in contrast to the suggestions from the BSAVA Manual of Rabbit Medicine, which states that proprioceptive positioning and placing responses are most useful and yield expected responses, while hopping, hemi-walking, and wheelbarrowing tests are difficult to perform [2]. The difference between their suggestions and our results may arise from various tips for handling animals, personal experiences, or variability in the temperament or behavior of animals from different geographic populations. The suggestions from the literature review by Vernau et al. were similar to our observations in general [4]. They stated that most rabbits do not return their paw to the normal position when performing proprioceptive positioning, placing responses may be difficult to elicit, and wheelbarrowing reactions can be elicited in healthy rabbits [4]. Although not specifically referring to rabbits, similar comments regarding variable postural reaction performance can be found in other small-animal neurology literature. de Lahunta et al. stated that the hopping reaction is the most reliable postural reaction test [14]. Regarding proprioceptive positioning (the paw placement test), most small animals immediately return the paw to its normal position. However, some neurologically healthy dogs may respond slowly or even fail to replace the paw if they do not bear weight on that limb [14]. Moreover, according to other authors, proprioceptive positioning is challenging in cats because they dislike having their feet manipulated during examination [12]. Although proprioceptive positioning and hopping reaction are both tests evaluating general proprioception, the literature has suggested that, along with motor function, the hopping response involves both conscious and subconscious proprioception. In contrast, the proprioceptive positioning test mainly involves conscious proprioception [15]. Although not proven, we speculate that this discrepancy between tests may result in tests more involved with conscious proprioception being more prone to abnormal responses, especially in species that may freeze or stiffen when manipulated.

Vernau et al. also mentioned that the hopping test is more sensitive than wheelbarrowing in detecting minor deficits [4]. Our personal experiences were similar. It is difficult to objectively assess whether the length, height, or pace of individual strides are normal or subtly decreased in the wheelbarrowing test in rabbits. By contrast, when performing hopping and hemi-walking tests, the lateral margin of the trunk can serve as a line of reference; therefore, we can be more accurate and confident in defining normal and abnormal responses in these tests. The normal response rates between the aforementioned tests were not significantly different in our study. We conclude that both tests can be performed during neurological examinations of rabbits. The hemi-walking test is safer and less stressful for rabbits because it does not require the rabbit to be lifted off the ground [13]. However, simultaneous assessment of the thoracic and pelvic limbs may be difficult, and the reaction may be obscured if the subject has long hair. In contrast, the hopping reaction allows for a more precise inspection of individual limbs. However, some rabbits tend to be uncooperative and try to jump away when held in the clinician’s hands.

Interestingly, high normal response rates were observed when performing the righting response in Warnefors’ (92%) and our (100%) studies [6]. The righting response is seldom performed in dogs or cats, and its significance has been discussed less. Its function allows animals to maintain a basic body posture of dorsal-side-up during movement or on the support surface [16]. It is unknown why, compared to other postural reaction tests, a relatively large percentage of rabbits responded normally to the righting reaction. It may be that the righting of body posture is a more primitive response for prey animals, possibly playing a role in fleeing predators if they are not successfully caught in the wild. However, the pathway involving the righting response is complex, and it is postulated that the righting response is controlled mainly by the brainstem. Musienko et al. demonstrated that rabbits decerebrated at the premammillary level could still display the righting response, although with slower movements [17]. The cerebellum and the spinal cord are likely to play a role in this response. In addition, it is unclear whether an asymmetrical righting response indicates lesions on the ipsilateral or contralateral side. In general, its value in lesion localization may be somewhat vague.

One limitation of this study was that all subjects underwent neurological examinations in an identical order. The rationale for this design is the large number of tests evaluated in the current study. We anticipated that we would be unable to recruit the large number of rabbits required if test-order randomization was applied. Nevertheless, the performance of individual tests may be influenced by the higher stress levels of the subject during the initial acclimation phase or a loss of patience during a prolonged examination. Although such a trend was not observed in our results, further prospective randomized studies are needed to assess these effects, possibly with a simplified examination list. One author examined all the subjects in the present study, and neither intra- nor inter-observer agreement was assessed. When conducting this study, the examiner already had 2–3 years of clinical experience in small-animal neurology; therefore, the intra-examiner variation was deemed minimal. To evaluate inter-examiner agreement, the subjects had to undergo a second round of examination. As the study aimed to assess many postural reaction tests or methods and then simplify the examination list, we thought it was not worth inflicting additional stress on the rabbits in the present study. Further research is warranted to evaluate inter-examiner agreement on the simplified exam list. Statistical analysis was not performed to determine the cut-off values for the tests in the present study. Instead, we arbitrarily drew a cut-off value of 90% for both feasibility and validity for all tests. We considered the suggested shortened rabbit neurological examination from Warnefors’ work [6], in which, although not clearly stated, most of the included tests had a successful practical performance and a neurological response of approximately 90% or more. The accuracy of our simplified list of postural reaction tests in neurologically ill patients remains unknown. Further research is needed to determine whether these tests can aid in accurate lesion localization in rabbits.

## 5. Conclusions

The hopping reaction (by holding and lowering the rabbit quickly towards the floor with only the tested limb touching the ground), hemi-walking, wheelbarrowing, and righting responses are feasible postural reaction tests that likely yield consistent and normal responses in healthy rabbits.

## Figures and Tables

**Table 1:** Methods for individual postural reaction tests.

Test	Method
Proprioceptive positioning	Flex the foot so that the dorsal surface of the paw is in contact with the floor
Hopping reaction—A	Hold the animal with its three feet on the surface
Hopping reaction—B	Hold the animal with only the tested limb touching the surface
Hopping reaction—C	Hold then lower the animal quickly towards the floor with only the tested limb touching the ground
Hemi-walking	Move the animal sideways
Wheelbarrowing	Support the animal under the abdomen with all the weight on the thoracic limbs
Extensor postural thrust	Support the animal under the thorax, and lower the pelvic limbs to the floor
Tactile placing	Support the animal under the thorax, bring the animal near the table, and mask its eyes with the other hand to prevent it from seeing the table surface
Righting response	Place and hold the animal in the lateral position, then release and observe its ability to rise

**Table 2:** The feasibility and normal response rate of individual postural reaction tests.

Test	Feasibility	Normal Response Right Side	Normal Response Left Side	*p*-Value ^†^
Proprioceptive positioning	TL: 97% (33/34) PL: 85% (29/34)	39% (13/33) NA	24% (8/33) NA	0.06 NA
Hopping reaction—A	100% (34/34) *	TL: 59% (20/34) PL: 18% (6/34)	TL: 68% (23/34) PL: 15% (5/34)	0.5 0.6
Hopping reaction—B	97% (33/34) *	TL: 94% (31/33) PL: 45% (15/33)	TL: 79% (26/33) PL: 52% (17/33)	0.06 0.7
Hopping reaction—C	97% (33/34) *	TL: 97% (32/33) PL: 88% (29/33)	TL: 97% (32/33) PL: 97% (32/33)	>0.9 0.4
Hemi-walking	97% (33/34) *	TL: 91%(30/33) PL: 76% (25/33)	TL: 91% (30/33) PL: 91% (30/33)	>0.9 0.06
Wheelbarrowing	100% (34/34)	94% (32/34)	94% (32/34)	>0.9
Extensor postural thrust	76% (26/34)	NA	NA	NA
Tactile placing	91% (31/34) *	TL: 13% (4/31) PL: 0% (0/31)	TL: 26% (8/31) PL: 0% (0/31)	0.1 >0.9
Righting response	97% (33/34)	100% (33/33)	100% (33/33)	>0.9

NA, not applicable; TL, thoracic limbs; PL, pelvic limbs. * Results for the thoracic and pelvic limbs are identical. ^†^ The normal response rates on the left and the right side were compared.

## Data Availability

The data presented in this study are available from the corresponding author upon reasonable request.
